# DPD Scintigraphy for diagnosis of amyloidosis in 1191 patients– a single centre experience

**DOI:** 10.1186/1750-1172-10-S1-O16

**Published:** 2015-11-02

**Authors:** David Hutt, Helen Mcphillips, Stephanie Mcknight, Julian Gillmore, Carol Whelan, Helen Lachmann, Ashutosh Wechalekar, Philip Hawkins

**Affiliations:** 1 University College London, National Amyloidosis Centre, NW3 2PF, London, UK

## Background

99mTechnetium labelled 3,3-diphosphono-1,2-propanodicarboxylic acid (99mTc-DPD) scintigraphy has been re-purposed in recent years as a sensitive non-invasive method for imaging transthyretin (ATTR) cardiac amyloid. We have routinely performed DPD scans since June 2010 at the National Amyloidosis Centre in London, and review here our findings in a large cohort of mostly British patients.

## Methods

Following administration of 99mTc-DPD, patients underwent whole body planar imaging three hours after injection followed by a cardiac SPECT-CT (single photon emission computed tomography with a low-dose, non-contrast CT scan). As of December 2014 we had performed 1454 DPD scans on a total of 1191 patients, which formed the basis of our cohort for analysis.

## Results

The most frequent amyloid types seen were light chain (AL) (n=277), wild-type ATTR (n=372) and hereditary ATTR (n=237). Of those AL patients with a positive scan (n=75), 24% had grade two or higher uptake compared to wild-type ATTR in which 95% of positive scans were grade two or three. A total of 22 different TTR variants were identified, most commonly Val122Ile (43%), Thr60Ala (25%) and Val30Met (16%); cardiac uptake at clinical presentation of patients with TTR Val122Ile and Thr60Ala were grade two or higher in 99% and 98% of cases respectively. Conversely, of the 16 TTR Val30Met patients with positive scans, four were grade one and the rest grade two. Interestingly, nine patients with the TTR Ser77Tyr mutation were studied, and despite a median left ventricular wall thickness of 15.5mm (range 13.5 – 20.0 mm), cardiac DPD tracer uptake was less than patients with other mutations who had an apparently similar amount of amyloid. Cardiac amyloidosis was ultimately excluded in 256 patients, in none of whom was there any cardiac uptake of DPD. Two patients with cardiac DPD uptake were found to have both AL and ATTR amyloid fibrils on cardiac biopsy.

Positive scans were obtained in three patients in whom cardiac biopsies were negative for amyloid. DPD scintigraphy was also performed on four patients who had received domino liver transplants from donors with familial amyloid polyneuropathy (one with Val30Met mutation). Cardiac uptake was seen in one patient (Phe33Val) who, eight years post transplantation, has now progressed to develop cardiac, peripheral and autonomic disease.

Extra-cardiac uptake of DPD was also noted, including soft tissue/skeletal muscle uptake in ATTR and AL patients, in some cases virtually obscuring cardiac tracer uptake. Lymph node and uptake in some cases of localized AL amyloidosis were also seen.

## Conclusion

DPD scintigraphy has proved to be a remarkably sensitive tool for the diagnosis and exclusion of clinically significant ATTR cardiac amyloidosis. Extra-cardiac uptake of DPD in amyloid deposits is an interesting phenomenon which warrants further investigation.

